# Visual feedback improves movement illusions induced by tendon vibration after chronic stroke

**DOI:** 10.1186/s12984-021-00948-7

**Published:** 2021-10-30

**Authors:** Salomé Le Franc, Isabelle Bonan, Mathis Fleury, Simon Butet, Christian Barillot, Anatole Lécuyer, Mélanie Cogné

**Affiliations:** 1 Rehabilitation Medicine Unit, CHU de Rennes, University Hospital of Rennes, 2, rue Henri Le Guilloux, 35000 Rennes, France; 2 Hybrid Unity, Inria, University of Rennes, Irisa, 6074 Umr Cnrs, Rennes, France; 3 Empenn Unity U1228, Inserm, Inria, University of Rennes, Irisa, 6074 Umr Cnrs, Rennes, France

**Keywords:** Stroke, Rehabilitation, Tendon vibration, Illusory movement, Virtual reality

## Abstract

**Background:**

Illusion of movement induced by tendon vibration is commonly used in rehabilitation and seems valuable for motor rehabilitation after stroke, by playing a role in cerebral plasticity. The aim was to study if congruent visual cues using Virtual Reality (VR) could enhance the illusion of movement induced by tendon vibration of the wrist among participants with stroke.

**Methods:**

We included 20 chronic stroke participants. They experienced tendon vibration of their wrist (100 Hz, 30 times) inducing illusion of movement. Three VR visual conditions were added to the vibration: a congruent moving virtual hand (Moving condition); a static virtual hand (Static condition); or no virtual hand at all (Hidden condition). The participants evaluated for each visual condition the intensity of the illusory movement using a Likert scale, the sensation of wrist’s movement using a degree scale and they answered a questionnaire about their preferred condition.

**Results:**

The Moving condition was significantly superior to the Hidden condition and to the Static condition in terms of illusion of movement (p < 0.001) and the wrist’s extension (p < 0.001). There was no significant difference between the Hidden and the Static condition for these 2 criteria. The Moving condition was considered the best one to increase the illusion of movement (in 70% of the participants). Two participants did not feel any illusion of movement.

**Conclusions:**

This study showed the interest of using congruent cues in VR in order to enhance the consistency of the illusion of movement induced by tendon vibration among participants after stroke, regardless of their clinical severity. By stimulating the brain motor areas, this visuo-proprioceptive feedback could be an interesting tool in motor rehabilitation.

*Record number in Clinical Trials:* NCT04130711, registered on October 17th 2019 (https://clinicaltrials.gov/ct2/show/NCT04130711?id=NCT04130711&draw=2&rank=1).

**Supplementary Information:**

The online version contains supplementary material available at 10.1186/s12984-021-00948-7.

## Introduction

Stroke is a public health problem and the leading cause of severe acquired disability in adults in developed countries. [1]. More than 60% of the stroke subjects present severe and constant upper limb motor injury without useful grip. [2]. Due to the loss of autonomy in daily life activities [3], the upper limb motor function recovery remains a major rehabilitation goal.

Tendon vibration inducing illusion of movement is an interesting tool to develop cortical excitability, neural plasticity and motor function after stroke [4]. The vibration can induce a powerful proprioceptive stimulation [5, 6] and create kinesthetic illusions [7] (i.e. some illusions of movement) probably by stimulating the brain motor areas [8] when accurate parameters are chosen (frequency around 80 Hz, tendon target) [9]. In motor rehabilitation, when the symptoms such as attention, cognitive and visual disorders are present, tendon vibration could be an attractive tool with the traditional rehabilitation.

We found in the literature that the illusory movement induced by tendon vibration could be increased by virtual visual cues [10]. A visual cue, congruent with the illusion of movement induced by tendon vibration, led to a higher kinesthetic illusion [11–13] compared to the absence of visual cues during the vibration period [14]. These studies targeted protocols using the “illusion mirror paradigm” [15] without VR interface and were conducted in healthy participants.

In a previous protocol, we studied the influence of congruent virtual visual cues on the illusion of movement induced by tendon vibration among healthy participants [16]. We determined that congruent visual virtual cues could significantly enhance the wrist illusion of movement during tendon vibration.

The aim of the present study was to test whether visual cues could improve the illusion of movement induced by tendon vibration among participants with chronic stroke. Knowing that participants who have had a stroke with upper limb paresis present brain lesions in the sensorimotor areas of their brain, we would like to explore whether illusions of movement induced by tendon vibration could be felt and whether visual cues may influence these illusions in participants with modified sensory inputs. Our research hypothesis was that visual feedback congruent to the illusion of movement would be also helpful for participants after chronic stroke, whatever the severity of the motor and sensory deficits nor the spasticity.

## Materials and methods

### Study design

We planned a monocentric randomized controlled pilot study in the Rehabilitation Unit of Rennes University Hospital in France. The Rennes University Hospital Center promoted the study and the Ethics Committee of Strasbourg University, France approved it on October 8^th^, 2019 (record number: 19/62-SI 19.07.05.46737). The participants received an information letter and each signed a written consent prior to testing. This study has been recorded in Clinical Trials under the record number NCT04130711, registered on October 17^th^ 2019 (https://clinicaltrials.gov/ct2/show/NCT04130711?id=NCT04130711&draw=2&rank=1).

### Participants

The participants were recruited in the Rehabilitation unit of Rennes University Hospital, France. A total of 20 participants with a mean age (± Standard Deviation) of 58.70 (± 12.57) years old (Min = 35, Max = 78) participated to the study, including 6 women (30%). All the participants fulfilled the following inclusion criteria: age between 18 and 80 years old, first unilateral ischemic or hemorrhagic hemispheric cerebral stroke, stroke occurring more than 6 months prior to enrollment (considered to be a period in which there is less expected recovery of the upper limb from conventional rehabilitation), mild to severe upper limb deficiency with Fugl-Meyer Assessment Upper Extremity (FMA-UE) score ≤ 60. Non-inclusion criteria were: ischemic or hemorrhagic damage to the posterior fossae, complete motor deficit of the upper limb, epicritical or proprioceptive anesthesia, comprehension disorders limiting participation in the study; participants deprived of freedom and with a legal incapacity were also excluded from this study.

## Experimental procedure

### Procedure

**Fig. 1:**
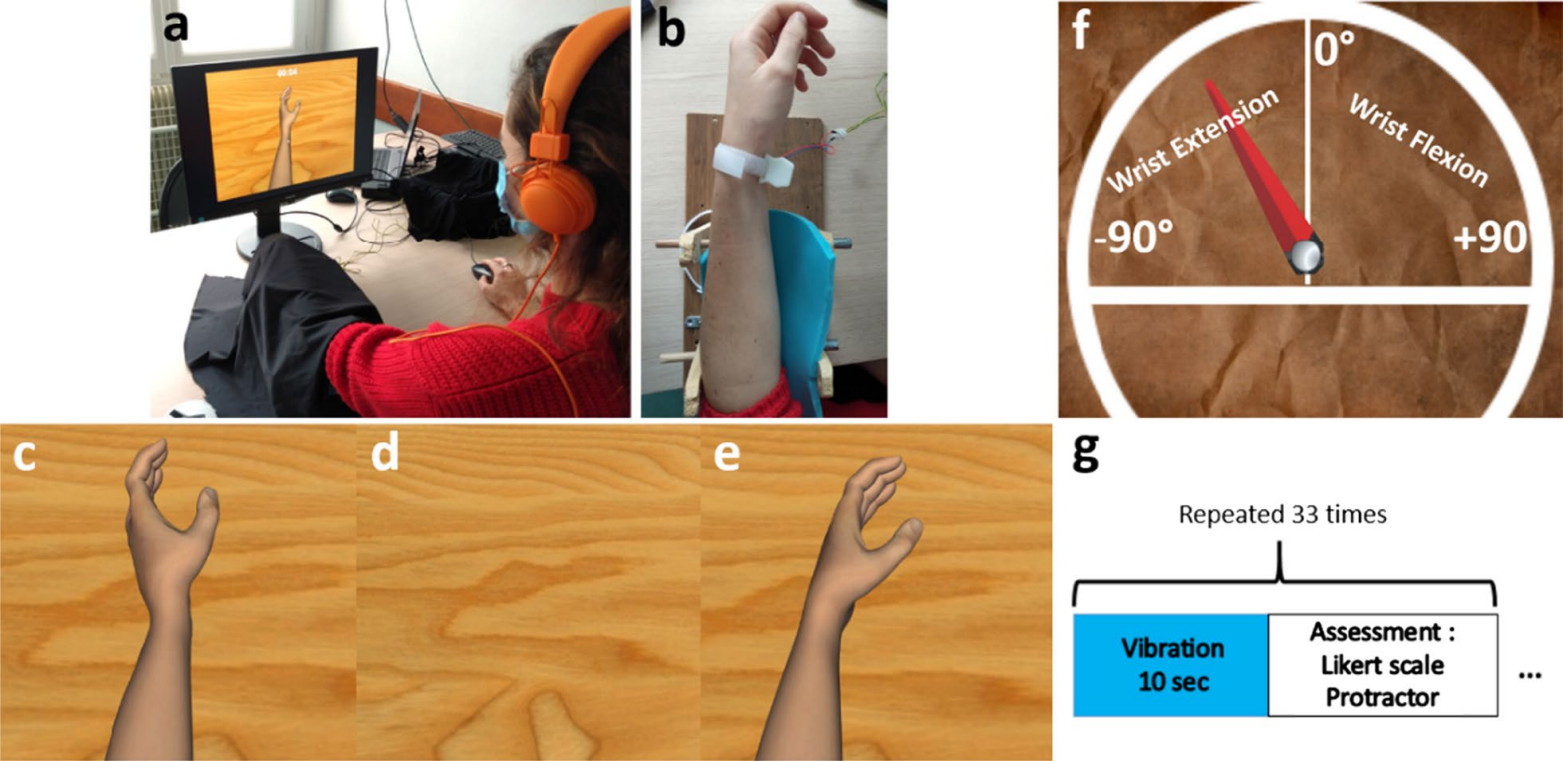
Illustrations of the equipment (example of the positioning of a left arm with a paresis after right stroke). **a**, **b** Installation of the vibrator. The forearm was covered with a cloth. **c**–**e** Presentation of the 3 virtual visual conditions (respectively Moving, Hidden, Static condition). The arrow is not visible during the experiment. **f** Protractor to measure the sensation of wrist’s displacement. « -90°» signifies a maximal wrist extension for the left upper limb. The description «values of degree» and « wrist extension, wrist flexion» are not seen by the participant during the trial. **g** Chronology of the trial

Before the experiment, we asked the participants about their laterality before and since the stroke by using an Edinburgh questionnaire. We also recorded several clinical aspects: pain in the disabled arm, articular limitation, epicritical and proprioceptive sensibility, arm spasticity, visual field, and motor function which was evaluated by using the FMA-UE [17]. The participants sat in an office chair in front of a computer screen. They placed their paretic arm on a cylindrical arm-holder (Fig. 1a, b), and their hand was covered with a black cloth, to not see it, with a vibrator applied on their flexor carpi tendon. (Fig. 1a). We applied tendon vibration during 10 s at the frequency of 100 Hz to induce some illusions of movement. For each vibration trial, one visual virtual cue among three was shown to the participants on the computer screen in a randomized order. The virtual cues could be: (1) a virtual hand moving in the same direction as the wrist extension (Moving condition) (corresponding to the expected feeling of illusion induced by the tendon vibration); (2) no hand at all with an empty screen (Hidden condition); (3) a static virtual hand (Static condition) (Fig. 1c–e). The participants answered two questions after each vibration trial: the intensity of illusion felt by using a Likert scale [18] from 1 to 7 (with 1 = no illusion at all; 4 = moderate intensity of illusion of movement; 7 = strong intensity of illusion of movement); the sensation of wrist movement in degrees by using a virtual protractor (Fig. 1f). Each participants tested 33 vibration trials (Fig. 1g), but the first three trials were not included in the analysis, considering the participants needed some time to focus on the vibration to feel illusions of movement and to make sure they had well understood the evaluation modalities. The participants were asked to fill out a questionnaire at the end of the protocol to get some information about their preferred visual conditions and subjective data on vibration comfort and feeling. Concerning the instructions, we explained orally to the participants the vibration modalities, without specifying which movement they could feel (hand, finger) nor in which direction it would occur. Then, we gave the same written instructions. We reminded the participants to focus on their upper limb sensations during the experiment.

### Visual feedback

**Fig. 2:**
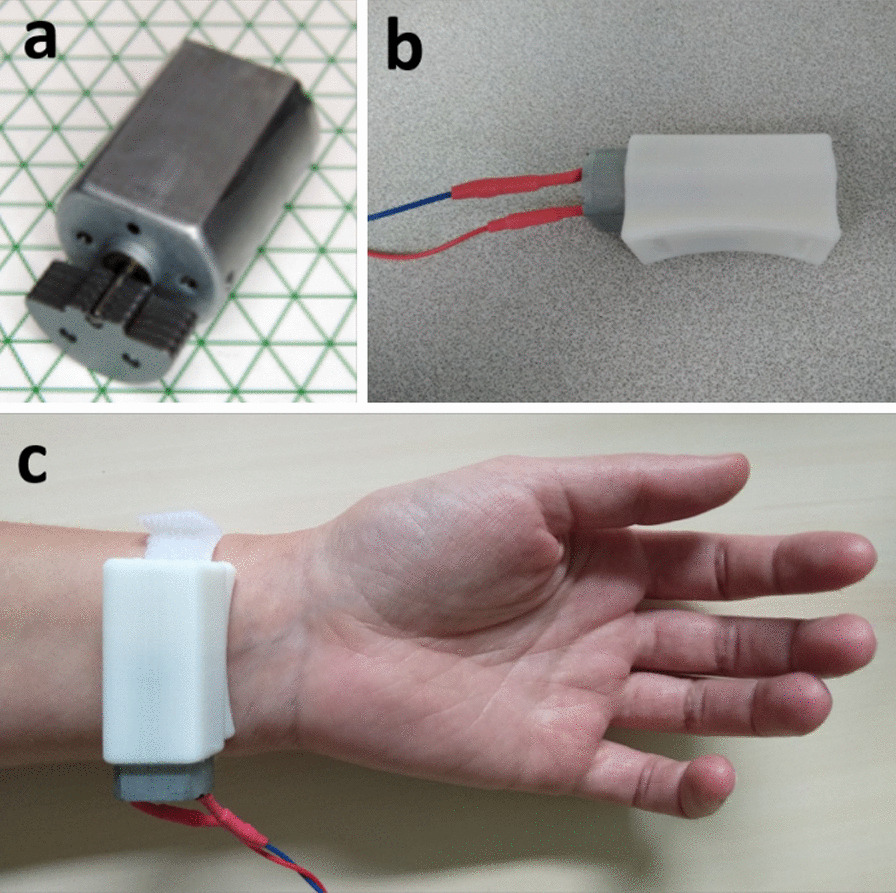
Pictures of the vibratory device UniVibe™. **a** Vibration motor. **b** Vibration device linked to the Arduino® and sound isolated. **c** Wrist positioning

The visual cues were displayed on a 17 inch-LCD monitor by using Unity software. The virtual hand appeared as natural generic skin upper limb avatar, in order to correspond to the participants’ own perspective. The virtual scene depended of the condition: (1) an extension of the non-dominant wrist at speed of 3 degrees per second, congruent with the illusory movement expected by the vibration on the flexor carpi tendon [8](Moving condition) (Fig. 1c); (2) an empty surface (Hidden condition) (Fig. 1d); a static hand (Static condition) (Fig. 1e). The device was available for both hands depending on the deficient side after the stroke.

### Vibratory device

The vibratory unit was a UniVibe™ Model 320–105 (Fig. 2). The device was composed of an actuator positioned on the flexor carpi tendon and was kept with an hook-and-loop fastener on the skin. The vibrator was inserted in a homemade sound box created by 3D print to protect the skin from the motor and to enhance the sensation of vibration. An Arduino® controlled the vibration motor. The vibration frequency was determined by the rotation of the mass. The diameter of the skin tactor was 25 mm. The parameters used were: frequency of 100 Hz, amplitude of 5G, voltage of 3.3 V [9, 19, 20] in order to elicit movement’s illusion.

### Collection of the data

Main measure consisted of the intensity of illusion of movement using the Likert scale [18] after each trial. Secondary outcome criteria were: the extent of movement experienced in degrees during each trial and their chosen visual condition. For the angle of motion, the participants could steer the needle of the protractor by using a computer mouse with their free functional hand. They positioned the needle from -90° to + 90° with all possible shades of degrees (Fig. 1f). If there was no illusions, they steered the needle on 0° (resting position). The protractor was available for both side. Due to the study design, we examined only the short-term effect in this study, and not the long-term effect. Data was collected in Data Archiving and Networked Services **(**DANS) database.

### Statistical analysis

We performed a descriptive analysis of all variables used in the study. We described qualitative variables with frequencies and their related percentages, as well as quantitative variables using the mean ± standard deviation.

The R software version 3.6.2 performed statistical tests. A non-parametric approach was used due to the repeated measures analysis of variance (ANOVA) which did not violate the assumption of sphericity according to Mauchly's test for the main judgment criterion (*i.e.*, the difference in intensity of illusion of movement: χ^2^ = 45.37, p < 0.001). We conducted a within-group analysis based on the Friedman tests comparing the 3 visual conditions and then we used the 2 by 2 conditions based on post-hoc tests (Wilcoxon signed rank test) corrected with Bonferroni. A Mann–Whitney test was used to perform others between-group comparisons.

## Results

**Fig. 3:**
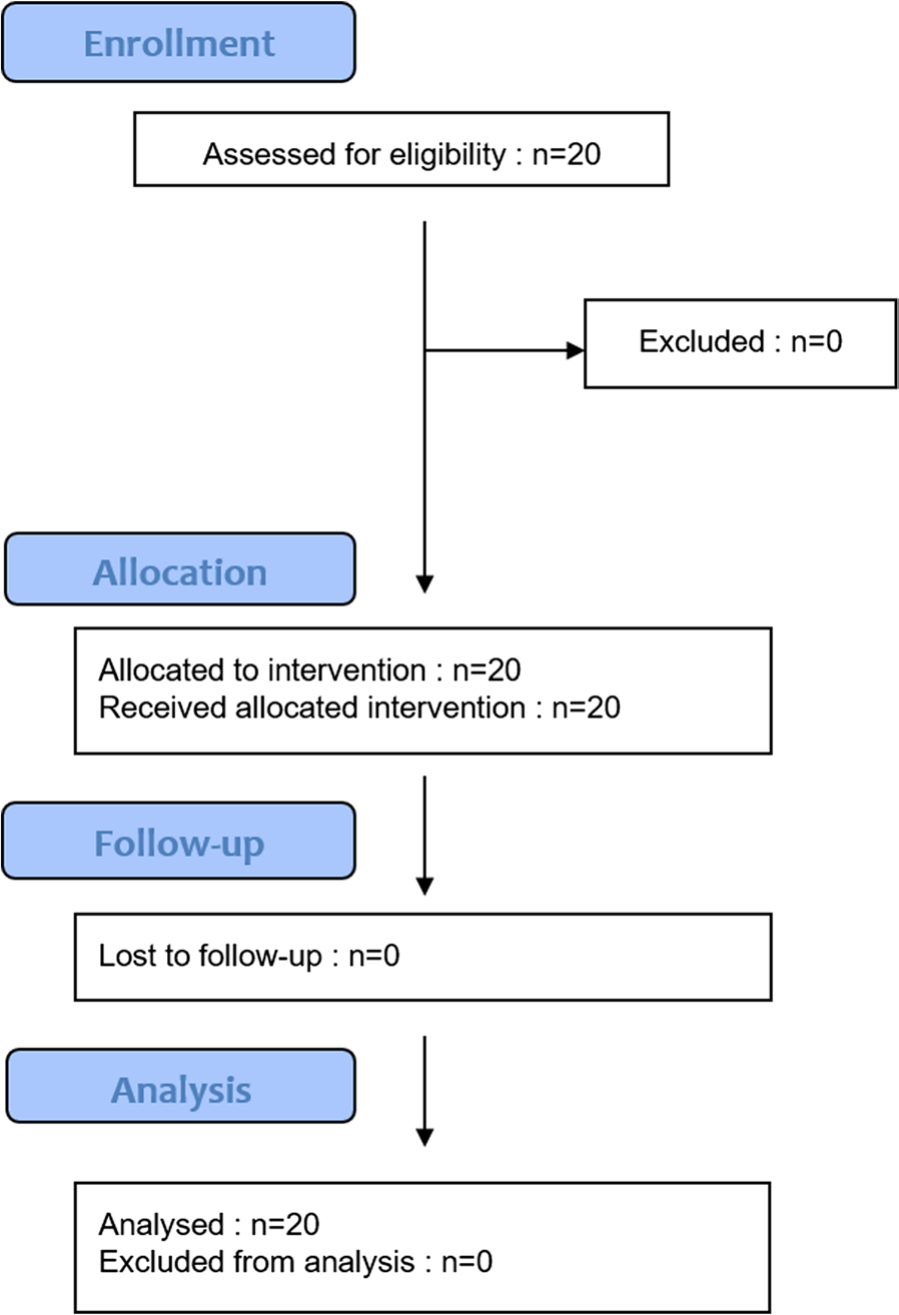
Flowchart of the experiment

The details of the flowchart of the experiment are presented in Fig. 3.

The clinical description of the population is detailed in Table 1.

**Table 1:** Clinical description of the participants

	Sex	Age	Time since stroke	Stroke	Side	FMA-UE	Sensibility	Spasticity
P1	M	63	483	I	R	58	N	N
P2	F	54	773	H	L	14	SN	Y*
P3	M	40	223	H	L	50	S	N
P4	F	61	3656	H	R	16	S	Y*
P5	F	40	303	I	L	47	SN	Y
P6	M	56	1040	I	R	53	SN	N
P7	M	47	896	H	L	56	S	Y
P8	M	59	837	H	R	59	S	Y
P9	M	41	1378	H	R	60	S	N
P10	M	66	1979	I	L	8	N	Y*
P11	M	68	315	I	L	49	SN	N
P12	M	52	403	H	L	26	M	Y*
P13	M	78	2122	I	L	6	SN	Y*
P14	M	35	2854	H	R	31	S	Y*
P15	M	69	837	I	L	49	M	N
P16	M	65	320	H	R	22	SN	Y
P17	F	69	1354	H	L	5	M	Y*
P18	M	74	437	I	R	43	M	N
P19	F	67	1122	I	R	60	M	N
P20	F	70	370	I	L	8	M	Y

*M*  male, *F* female, *I* ischemic, *H*  hemorrhagic, Age in years old, Time since stroke in days. Stroke side: *R* right hemisphere, *L* left hemisphere

*FMA-UE* Fugl-Meyer assessment upper extremity, motor item (66 points)

Sensibility item: *N* normal, *SN* subnormal, *M*  moderate disorder, *S* severe disorder

Spasticity item: *N * no, *Y* yes, * = severe spasticity with joint deformation

### Intensity of the illusion of movement

**Fig. 4:**
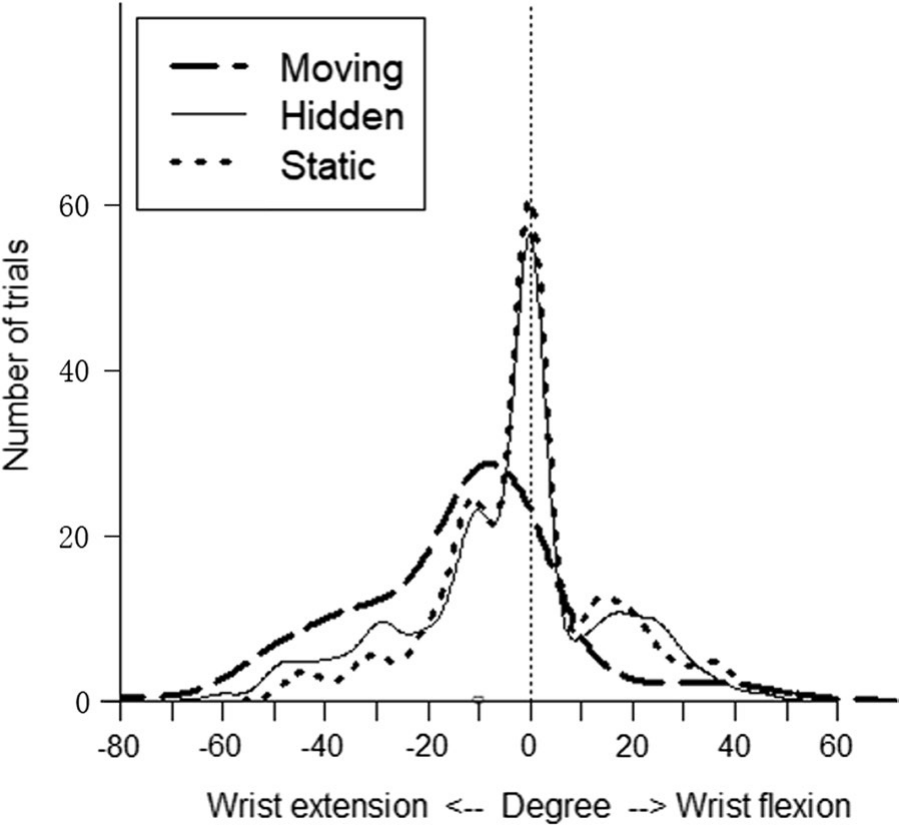
Boxplot representing the intensity of illusion of movement. Intensity of illusion of movement experienced in each condition, averaged across all participants (respectively for Moving, Hidden, Static condition). The mean is indicated by dots

The mean (± SD) Likert ranking was respectively 3.40 (± 1.67) for the Moving condition, 2.98 (± 1.78) for the Hidden condition and 2.79 (± 1.70) for the Static condition (Fig. 4), averaged in all participants. A Friedman test showed a statistically significant difference between the 3 visual conditions concerning the Likert scale ranking (χ^2^ = 45.37, p < 0.001). Post-hoc analysis showed that the Moving condition induced a higher intensity of illusion of movement than the Hidden condition (p < 0.001) and the Static condition (p < 0.001). We did not find any difference between the Hidden condition and the Static condition (p = 0.06).

Then we used a Mann–Whitney test to make other between-group comparisons. We split the group in two, depending on their upper limb dysfunction (severe or moderate dysfunction), and we compared the data concerning the intensity of the illusion of movement (in the Moving condition). We did not find any significant difference between the group with severe motor function disability (FMA-UE ≤ 30, n = 8, FMA-UE mean = 13.13) compared to that with moderate motor function disability (FMA > 30, n = 12, FMA-UE mean = 51.25) (p = 0.99).

Then, by separating right- and left-hemispheric stroke (right: n = 9), we found a significant difference concerning the intensity of illusion of movement (p < 0.01) with higher illusions in the right stroke group. Further analysis showed that the upper limb motor function (measured by FMA-UE) was better in the right stroke group (FMA-UE mean = 44) compared to that of the left one (FMA-UE mean = 28).

We did not find higher illusions of movement among participants with normal to moderate sensibility disorders compared to those with severe disorders (n = 14 and n = 6 respectively) (p = 0.31).

Concerning the effect of spasticity, there was no significant difference between participants with severe spasticity (n = 12) and weak to moderate spasticity (n = 8) concerning the intensity of illusion of movement (p = 0.83).

### Sensation of wrist’s extension

**Fig. 5:**
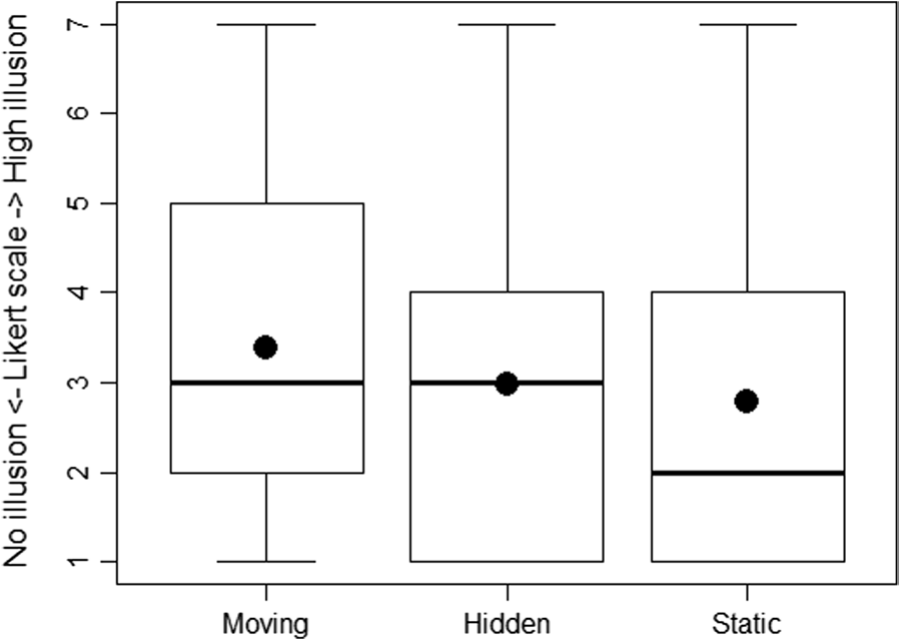
Frequency of sensation of wrist extension. The smoothed histogram depicting the frequency of sensation of wrist movement in each condition averaged across the participants. The zero-degree axis is represented by the vertical line (no illusion). The figure represents the distribution of the values from the protractor, with negative degrees for wrist extension and positive degrees for wrist flexion

The mean value (± SD) of the sensation of wrist’s extension of the participants in degrees was respectively -13.80 (± 20.91) for the Moving condition, -4.40 (± 19.18) for the Hidden condition and -1.02 (± 17.12) for the Static condition, averaged in all participants (Fig. 5). A Friedman test showed a statistically significant difference between the three conditions (χ^2^ = 69.90, p < 0.001). Post-hoc analysis showed that the Moving condition induced a higher sensation of wrist’s extension than the Hidden condition and the Static condition (p < 0.001). There was no significant difference between the Hidden condition and the Static condition concerning the sensation of wrist’s movement (p = 0.08). We performed a Spearman correlation between the intensity of illusion of movement and the sensation of wrist’s extension. We found a moderate significant negative correlation between these 2 parameters for the Training condition (Spearman’s correlation coefficient rho = -0.61, p < 0.001).

### Subjective reports of the participants

At the end of the protocol, the participants answered a questionnaire. Among the 20 participants included, 6 of them (30%) had already had a small experience of illusion of movement induced by neck muscle vibrations (used in clinical practice for reducing unilateral neglect). The Moving condition was the participants’ preferred condition to enhance the illusion of movement (n = 14, 70%), then the Hidden one (n = 1, 5%). Some participants did not prefer any of the 3 conditions (n = 5, 25%) and no participants preferred the Static condition. Concerning the type of illusion of movement, they mainly felt a wrist’s extension (n = 7, 39%), then a wrist’s supination (n = 4, 22%), then a wrist’s flexion (n = 3, 17%) or an ulnar’s deviation (n = 3, 17%) and a fingers’ extension (n = 1, 6%). Two participants (P11 and P15) did not feel any illusion of movement at all. During the experiment, all the participants reported a correct feeling of vibration (n = 20, 100%), and no participants declared any uncomfortable feeling of paresthesia or itching.

## Discussion

Our experiment studied whether visual cues could improve the illusion of movement induced by tendon vibration among participants with chronic stroke. We found that the illusion of movement was higher in participants with chronic stroke when the visual cues were congruent to the illusion induced by the tendon vibration, compared to incongruent cues. The results highlighted that the Moving condition significantly increased the intensity of illusion of movement (Fig. 4), sensation of wrist movement (Fig. 5) and visual comfort for the participants compared to the Hidden and Static condition. We also found a correlation between the intensity of illusion of movement and the sensation of wrist’s extension.

Contrary to the literature and the results of a similar previous study with healthy participants [16, 21, 22], the Hidden and the Static conditions were comparable in terms of sensation of wrist displacement and procured a weak intensity of illusion of movements in the participants. We considered that the Static condition was an “incongruent” stimuli, whereas the Hidden condition was a “neutral” stimuli and the Moving condition a “congruent” stimuli. In the literature, we found that a tendon vibration during blindness could allow illusion of movement [21]. We compared in a previous study including healthy participants a neutral stimuli (corresponding to blindness) to congruent or incongruent stimuli [16]. We found that a congruent condition (i.e. a moving condition) was significantly higher than a neutral condition (i.e. a hidden condition) and an incongruent condition (i.e. a static condition). The neutral condition was also significantly higher than the incongruent condition in term of illusion of movement. The results in healthy participants differed that those that we present in the current study. These results can be due to the small sample size and a lack of power in our study. However, our sample size of 20 was higher than the protocols found in the literature which was around 15 participants. Moreover, among the participants, several trials in the Hidden and Static conditions did not induce any illusion of movement at all. We know that stroke can cause motor and sensibility disorders, with a decrease in the ability to produce illusions of movement [23], similar to neurological diseases such as cerebral palsy or dystonia [24, 25]. Few studies focused on the evaluation of the illusion of movement induced by tendon vibration in stroke patients. Beaulieu et al. (2020) tested a procedure to evaluate the illusion of movement induced by tendon vibration in healthy participants and some chronic stroke patients. They showed that the perceived illusion of movement was significantly decreased on paretic side in stroke subjects compared to the non-paretic side or compared to healthy participants. This study focused on ankle vibration but without visual support [23]. Another study from Beaulieu et al. (2017) showed that tendon vibration combined with other proprioceptive stimulation used in a population of chronic stroke subjects may influence the motor cortical areas plasticity injured after stroke [26].

The illusions of movement seemed weaker in the stroke population compared with healthy participants of our previous study. In healthy subjects, the kinesthetic illusions seems to enhance the cortical activity in sensorimotor areas and the propriomotor loop [27, 28]. The participants in the study had undergone motor cortical or subcortical lesions, which can cause low illusions of movement.

Our results showed the advantages of using congruent visual cues in a population of participants with stroke to enhance their illusions of movement, when tendon vibration with no visual cues (corresponding to Hidden condition) or incongruent cues (corresponding to Static condition) did not allow a sufficient illusion of movement (Fig. 4). The literature proved the importance of the embodiment to feel the best illusion, requiring a good correspondence between the visual or VR environment and the properties of the real human body [29, 30].

Interestingly, concerning the population studied, we found that the illusion of movement was felt regardless of the severity of the upper limb’s characteristics of the participants in terms of motor function, sensibility and spasticity (Figs. 4, 5, Table 1). We divided the stroke patients into "mild group" and "moderate or severe group" by their FMA scores. We choose 30 FMA points cut-off to classify the severity of the upper limb deficiency, without official reference. In clinical practice, a FMA-UE score < 30 reflects a non-functional upper limb, that we considered as a severe deficiency. Beyond this score, the subjects can partially use their upper limb for some useful movements in daily-life activities. Even among the most severe profiles of disability, illusions of movement were present. We can now consider the association of tendon vibration and congruent visual cue as a useful tool to stimulate stretching, proprioception and brain plasticity among this population of subjects after chronic stroke with moderate to severe disabilities.

Subjects with a right hemispheric stroke can develop attentional disorders that can induce difficulties in feeling body illusions. Surprisingly, we found that the participants’ subgroup with a right hemispheric stroke felt higher illusions of movement that the subgroup with a left hemispheric stroke. We found in the literature robust results concerning the role of the right hemisphere in kinesthetic processing and perception of limb movement. Naito and al. (2005, 2007) proved the dominance of the right hemisphere activation during tendon vibration in left and right upper limb among healthy participants [31, 32]. During illusions of movement, the activated structures are larger and more intense in the right hemisphere than in the left hemisphere. These findings can explain the preservation of movement illusions after a right hemispheric stroke when several other brain structures involved in the integration of kinesthetic information can be activated. On the contrary, only a small area of structures are involved in the left hemisphere during movement illusions [27], which could all be easily injured after a stroke.

Among the participants, two (P11 and P15) did not feel any illusion of movement. Their clinical presentation was rather similar, around 70 years old, moderate upper limb motor disability and sensibility disorders without spasticity (Table 1). They both suffered from an ischemic left stroke, in the cerebral anterior area (P11) or at the junction between cerebral anterior and middle cerebral artery (P15). We found in the literature that prefrontal medial regions could be involved in the awareness of illusory movements, and that they could have been altered after stroke in these participants [33, 34]. Other studies found equal results concerning the lack of illusions of movement among patients after stroke and even in healthy participants [23, 35].

We did not focus on the after effect in our experiment. However, some participants mentioned spontaneously the existence of an after effect sensation during the experiment after the vibration.

Our study is part of a broad project to study the interest of the proprioceptive stimulation included in a Neurofeedback protocol to upper limb motor rehabilitation in post-stroke population. The results of the current study allowed us to use the tendon vibration tool with a congruent visual cue for future studies. Further developments will combine the upper limb tendon vibration associated with congruent visual cues as feedback in EEG Neurofeedback study for motor rehabilitation (Additional file 1: Table S2).

## Study limitations

We did not perform a power calculation, which can induce discussion about the statistical results. It was a pilot study including more patients that the other protocols on the same topic [23]. We made a within-design analysis, and the between-design was not applicable. Ten trials were analyzed in each condition for all the participants and it was not sufficient to make a between-design. Another limit is that the outcomes are restricted to subjective clinical data alone.

Then, we may have underestimated the illusions of movement among the participants. The virtual protractor was designed to describe wrist extension or flexion. Sometimes, the participants felt other kinds of movement illusions, not corresponding to extension or flexion, and the participants answered “0°” on the protractor, not being able to describe any other movement. We only assessed in this current study the ability to the stroke participants to feel illusion of movement, whatever the kind of illusion, depending of the visual conditions. In Fig. 5, some vibration trials released wrist flexion sensation in the Moving and Hidden condition. It could be due to the tonic vibe reflection. We observed this effect in participants with spasticity but also participants without spasticity in our experiment. Nevertheless, we did not quantify the tonic vibe reflection in this sense.

Finally, we also must take into account the population studied here, i.e. participants after stroke, who in addition to motor deficits, may present some visuospatial and cognitive disorders making the analysis of movement and its direction more difficult than in healthy participants.

## Conclusion

Our perspective was to determine the more efficient combination of VR and tendon vibration for obtaining illusion of movement in order to further develop new tools to motor post-stroke rehabilitation. In conclusion, the results highlighted that visual virtual cues could improve the illusion of movement induced by tendon vibration when they appeared congruent to the tactile stimulation among participants with stroke. It demonstrates the importance of the embodiment using visuo-proprioceptive stimulation. Moreover, it suggests that most stroke participants, even with severe profiles in terms of motor function, sensory impairments and spasticity could use this visuo-proprioceptive tool in rehabilitation.

### Clinical messages

- TV with congruent visual cues can be combined to increase the feeling of illusion of movement
- These tools can be used in stroke rehabilitation

## Supplementary Information

**Additional file 1: Table S2. **Summary of data for each patient.

## Data Availability

Data of the study are available in DANS repository: https://easy.dans.knaw.nl/ui/datasets/id/easy-dataset:198452.

## Abbreviations

ANOVA: Analysis of variance
FMA-UE: Fugl-Meyer Assessment Upper Extremity
VR: Virtual Reality
