# Supplementary material for: Visual feedback improves movement illusions induced by tendon vibration after chronic stroke
Source: J Neuroeng Rehabil. 2021 Oct 30;18:156. doi: 10.1186/s12984-021-00948-7 (PMC8556973; doi:10.1186/s12984-021-00948-7)
Supplement: Supplementary file 1 — Additional file 1: Table S2. Summary of data for each patient. [file 12984_2021_948_MOESM1_ESM.docx]

**Additional file 1**

***
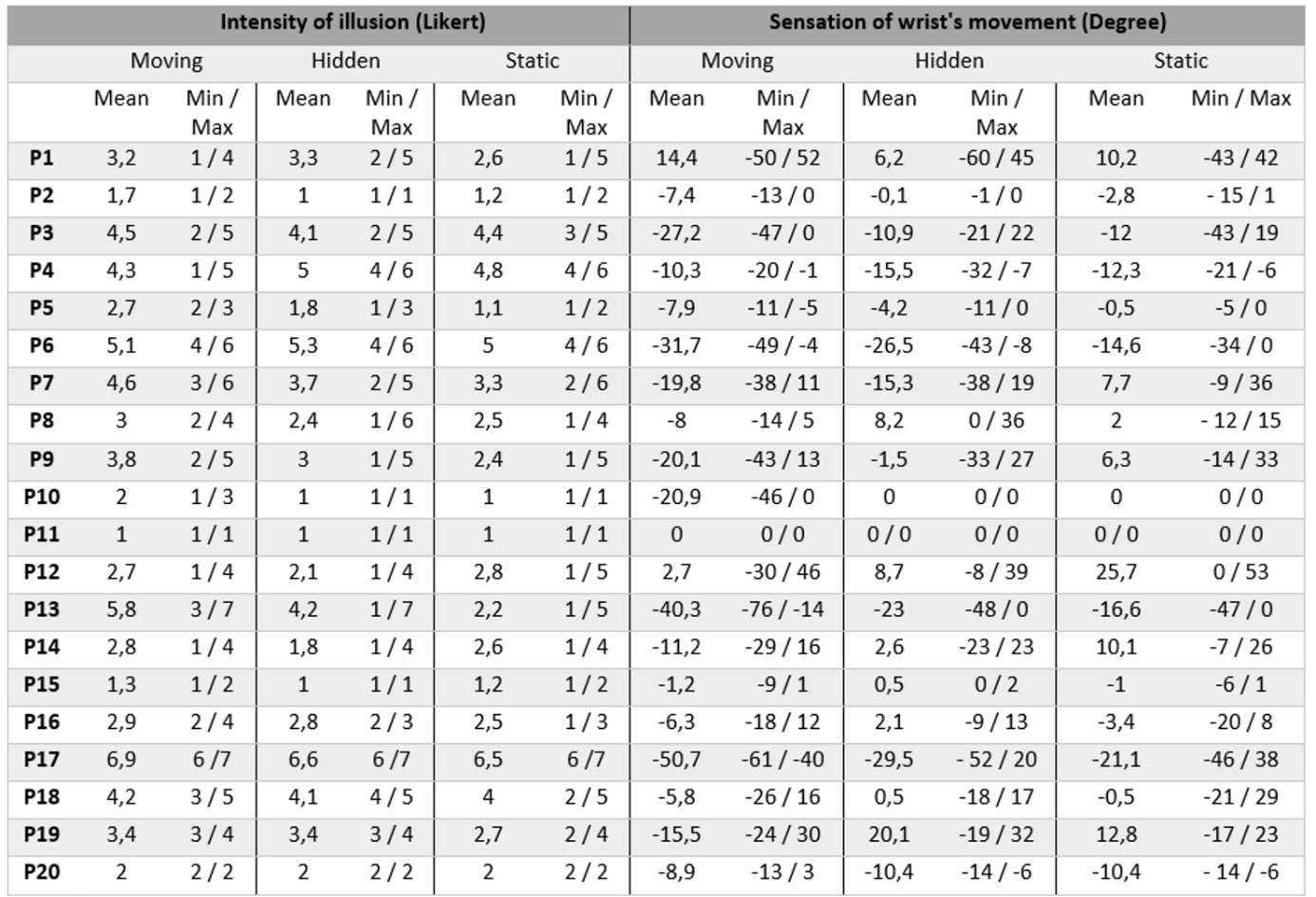
***

**Table S2. Summary of data for each patient**

Results for each patient (from P1 to P20) on the intensity of illusion of movement (based on Likert scale) and the sensation of wrist movement (based in degrees), concerning each condition (Moving, Hidden, Static).
